# Metabolite profile comparison of a graft chimera ‘Hongrou Huyou’ (*Citrus changshan-huyou* + *Citrus unshiu*) and its two donor plants

**DOI:** 10.1186/s12870-019-2173-4

**Published:** 2019-12-26

**Authors:** Min Zhang, Luyang Jing, Qun Wu, Kaijie Zhu, Fuzhi Ke, Jianguo Xu, Siqing Zhao, Gang Wang, Chi Zhang

**Affiliations:** 1 State Key Laboratory of Subtropical Silviculture, Zhejiang A& F University, Hangzhou, 311300 China; 2 Quzhou Technical Extension Station for Cash Crops, Quzhou, 324000 China; 3 Key Laboratory of Horticultural Plant Biology (Ministry of Education), Huazhong Agricultural University, Wuhan, 430070 China; 4 Citrus Research Institute of Zhejiang Province, Huangyan, 318020 China; 5 Changshan Huyou Research Institute, Quzhou, 324000 China

**Keywords:** Citrus, Periclinal chimera, Metabolites, Volatiles, Carotenoids

## Abstract

**Background:**

Chimeras synthesized artificially by grafting are crucial to the breeding of perennial woody plants. ‘Hongrou Huyou’ (*Citrus changshan-huyou* + *Citrus unshiu*) is a new graft chimera originating from the junction where a *Citrus changshan-huyou* (“C”) scion was top-grafted onto a stock Satsuma mandarin ‘Owari’ (*C. unshiu*, “O”). The chimera was named OCC because the cell layer constitutions were O for Layer 1(L1) and C for L2 and L3. In this study, profiles of primary metabolites, volatiles and carotenoids derived from different tissues in OCC and the two donors were investigated, with the aim of determining the relationship between the layer donors and metabolites.

**Results:**

The comparison of the metabolite profiles showed that the amount and composition of metabolites were different between the peels and the juice sacs, as well as between OCC and each of the two donors. The absence or presence of specific metabolites (such as the carotenoids violaxanthin and *β*-cryptoxanthin, the volatile hydrocarbon germacrene D, and the primary metabolites citric acid and sorbose) in each tissue was identified in the three phenotypes. According to principal component analysis (PCA), overall, the metabolites in the peel of the chimera were derived from donor C, whereas those in the juice sac of the chimera came from donor O.

**Conclusion:**

The profiles of primary metabolites, volatiles and carotenoids derived from the peels and juice sacs of OCC and the two donors were systematically compared. The content and composition of metabolites were different between the tissues and between OCC and the each of the two donors. A clear donor dominant pattern of metabolite inheritance was observed in the different tissues of OCC and was basically consistent with the layer origin; the peel of the chimera was derived from C, and the juice sacs of the chimera came from O. These profiles provide potential chemical markers for genotype differentiation, citrus breeding assessment, and donor selection during artificial chimera synthesis.

## Background

Plant chimeras are plants composed of cells with more than two genotypes. According to the theory of ‘Tunica-Corpus’, the shoot apical meristem (SAM) of dicotyledonous plants is composed of three cell layers, namely, L1, L2 and L3, from the outermost layer [1]. In citrus fruits, the juice sacs and epidermal pericarps are derived from L1; the color and aroma of the fruit rind, seeds and segment walls are developed from L2; vascular bundles are produced by L3; and fruit shape is determined by L2 and/or L3 [2]. To date, there have been some reports on the discovery and identification of citrus chimeras. Zhou et al. found that the interaction between cells derived from different genotypes caused DNA mutation in the periclinal chimera fruits NFF (L1-L2-L3 = N-F-F) and FNN [2]. Wu et al. found that the fruit characteristics of the chimera Ekuliku were inconsistent with the source donor and that cross-sectional structure of the blade of the chimera was quite different from that of the two donors [3]. Zhang and his colleagues investigated two citrus chimeras named ‘Zaohong’ navel orange [4] and ‘Hongrou Taoye’ orange [5]; both two chimeras were produced from the donors sweet orange (*Citrus sinensis*) and Satsuma mandarin. The stomatal density and the flesh aroma of the chimera fruits in their studies were not consistent with those of the source donor; the chimera fruits showed combined characteristics of both donors [4, 5]. Since these variations in morphology and DNA mutation level occurred in plant chimeras, the accumulation patterns of metabolites in tissues and/or the cell interactions in chimeras warrant further study.

Citrus fruits are highly valued for their nutrient components, and many studies have investigated the metabolites in oranges (*C. sinensis*), mandarins (*C. reticulata*), pummelos (*C. grandis*) and grapefruits (*C. paradisi*) [6–9]. Primary metabolites, such as sugars and organic acids, are a diverse class of organic compounds that are essential for plant growth and internal quality [10]. For example, a high content of citric acid coinciding with a high level of free amino acids (especially proline) may be a reason that the shelf life of lemon is longer than that of other citrus [11]. Volatiles include several important secondary metabolites and have received extensive attention due to their marked health-promoting effects and high commodity value. *d*-Limonene is a dominant volatile in citrus and specifically protects against breast and rectal cancer [12]. Linalool and linalyl acetate have been used as anti-inflammatory agents [13], and rearrangements of germacrene D eventually produced some natural compounds [14]. Carotenoids are complex and abundant molecules in citrus fruits [15]. Some carotenoids containing *β*-ring moieties are precursors of vitamin A, which are highly beneficial to chronic disease and cancer prevention [16]. Carotenoid biosynthesis and regulation in citrus fruits have been extensively studied [17–19], and these reported were helpful for our analysis of carotenoid expression in chimeras.

**Fig. 1:**
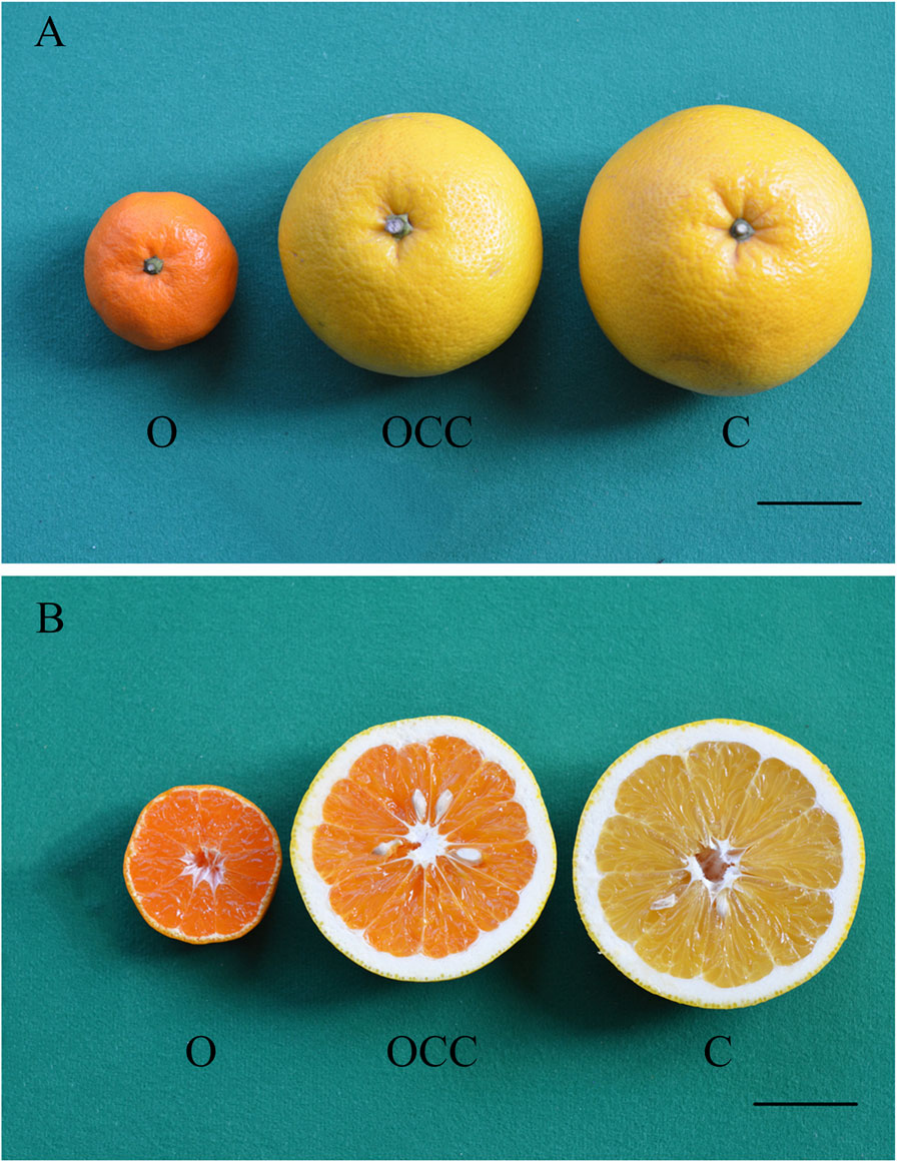
Fruit morphology of ‘Hongrou Huyou’ (OCC) and its donor plants were harvested at the full ripening stage External appearance (A) and transverse section appearance (B) of ‘Owari’ satsuma mandarin (O), OCC (the chimera) and ‘Changshan Huyou’ (C), from left to right, are shown. The bars of the external and transverse sections are both 5.0 cm.

Graft chimeras are derived from an adventitious shoot at the graft junction and comprised of two distinct genotypes or different species [20–23]. A new graft chimera, ‘Hongrou Huyou’ (*Citrus changshan-huyou* + *C. unshiu*) was originated from the junction of the scion ‘Changshan Huyou’ (*C. changshan-huyou*, abbreviated “C”) and the stock ‘Owari’ Satsuma mandarin (*C. unshiu*, abbreviated “O”). This chimera remained yellow-skinned, similar to C, but gained the dark orange juice sacs observed in O (Table 1, Fig. 1). Additionally, the chimera combined the specific DNA bands of the two donors in the nuclear, chloroplast and mitochondrial genomes through simple sequence repeat (SSR) amplification. Therefore, the chimera was assumed to be OCC because L1 was derived from O, while L2/ L3 were derived from C (data not shown).

**Table 1:** The cultivars used in this study and their morphological traits

No.	Cultivars	Scientific name	Abbreviation	Peel color	Juice sac color
1	‘Owari’ satsuma mandarin	*C. unshiu*	O	Orange	Dark orange
2	‘Hongrou Huyou’	*C. unshiu + C. changshan-huyou*	OCC	Yellow	Dark orange
3	‘Changshan Huyou’	*C. changshan-huyou*	C	Yellow	Light yellow

Note: Peel color and juice sac color were taken in fully mature period (collection period December 2017), see Fig. 1 for details

In this study, the profiles of primary metabolites, volatiles and carotenoids at the maturation period were investigated in the peels and juice sacs of the chimera and the two donors, and the correlation of metabolite accumulation between the chimera and each donor plant was analyzed to reveal the contributions of the donor plants to the different layers.

## Results

### Primary metabolites in OCC and its donors

As shown in Table 2, twenty-one primary metabolites were identified in peels. Based on statistical analysis, the peels of OCC (OCP) shared more similarities with the peels of C (CP) than the peels of O (OP) in these profiles. Among them, 4-aminobutanoic acid, shikimic acid and palmitic acid were exclusively detected in OCP and CP, suggesting that these 3 compounds in OCP were only produced by CP. In contrast, sorbose was specific to OP, with no detectable levels in OCP and CP. Both OCP and CP possess higher concentrations of acids and lower contents of total sugars; however, OCP contains middle contents of alcohols. Overall, the total metabolite content present in OCP was significantly lower than that in either of the donors.

**Table 2:** Primary metabolite profiles (μg g-1 FW) in the peels of OCC and its donor plants

No.	Primary metabolites (μg g^− 1^)	OP	OCP	CP
Organic acids				
1	Carbamic acid	8.23 ± 0.45c	205.82 ± 14.44a	173.78 ± 9.83b
2	Cyclohexaneacetic acid	0.56 ± 0.09c	2.28 ± 0.41b	3.35 ± 0.32a
3	Malic acid	92.87 ± 9.42c	211.84 ± 8.73b	280.92 ± 12.48a
4	Quininic acid	273.14 ± 11.78b	341.09 ± 25.51a	354.57 ± 47.87a
5	2-Ketoglutaric acid	64.77 ± 6.22a	35.08 ± 2.73b	41.24 ± 3.70b
6	4-Aminobutanoic acid	nd	42.49 ± 3.71a	24.26 ± 7.77b
7	Shikimic acid	nd	11.21 ± 1.44a	11.16 ± 1.06a
8	Palmitic acid	nd	29.14 ± 2.07a	31.97 ± 1.54a
	Sum	439.57 ± 27.97b	878.94 ± 59.03a	921.27 ± 84.57a
Sugars				
9	Xylose	215.09 ± 3.73a	79.35 ± 6.14c	112.00 ± 5.65b
10	Mannose	24,997.79 ± 1538.92a	11,139.04 ± 654.61c	14,008.21 ± 659.76b
11	Galactose	6961.91 ± 512.69a	2910.33 ± 191.21c	3885.98 ± 176.32b
12	Fucose	5.83 ± 0.60c	12.82 ± 1.55a	9.50 ± 0.92b
13	Fructose	20,950.50 ± 1276.05a	9533.23 ± 501.14b	10,982.41 ± 464.13b
14	*d*-Psicose	246.44 ± 11.15a	47.54 ± 9.82c	88.83 ± 6.39b
15	Turanose	91.76 ± 9.73a	25.64 ± 2.07b	33.00 ± 2.89b
16	Sucrose	16,576.74 ± 471.54a	7352.21 ± 162.93c	8777.85 ± 359.66b
17	Myo-Inositol	1102.16 ± 76.96a	1035.31 ± 59.25ab	965.58 ± 39.84b
18	Sorbose	160.32 ± 4.82a	nd	nd
	Sum	71,308.53 ± 3906.19a	32,135.47 ± 1588.70b	38,863.34 ± 1715.57b
Alcohols				
19	Glycerol	125.80 ± 13.79c	247.62 ± 32.8b	303.96 ± 32.17a
20	Scyllo-Inositol	54.75 ± 4.92c	169.21 ± 4.08b	291.89 ± 14.46a
	Sum	180.56 ± 18.72c	416.84 ± 36.89b	595.85 ± 46.63a
	Total	71,928.66 ± 3952.88a	33,431.25 ± 1684.62c	40,380.46 ± 1645.96b

Notably, some particular chemical characteristics were observed in OCP. Among acid profiles of the three samples, the level of carbamic acid was the highest in OCP; but the contents of sugar compositions (except of fucose, fructose and turanose) were the lowest in OCP.

Eighteen primary substances, listed in Table 3, were detected among the juice sacs of O (OJ), OC (OCJ) and C (CJ). In the present study, no significant differences were found in the total primary metabolites between OCJ and the juice sacs of the two donors. Interestingly, 5 metabolites (quininic acid, xylose, arabinose, turanose and scyllo-inositol) were significantly different in OCJ and in the juice sacs of the two donors. Among the three cultivars, the levels of arabinose and quininic acid in OCJ were the highest and the lowest, respectively. The remaining 3 metabolites in OCJ were significantly different from those in the two donors. In addition, 8 metabolite profiles in OCJ were consistent with one or two of the donors. However, these profiles actually showed more similarities with O; for example, 4-aminobutanoic acid, palmitic acid and allose were present in both OCJ and OJ but were not present in CJ. Conversely, sorbose was only present in CJ and was undetectable in OCJ and OJ.

**Table 3:** Primary metabolite profiles (μg g^−1^ FW) in the juice sacs of OCC and its donor plants

No.	Primary metabolites (μg g^− 1^)	OJ	OCJ	CJ
Organic acids				
1	Oxalic acid	22.67 ± 0.90a	nd	19.16 ± 3.99a
2	Malic acid	206.33 ± 21.44b	172.40 ± 8.68b	363.95 ± 60.83a
3	4-Aminobutanoic acid	10.36 ± 1.00b	16.01 ± 4.16a	nd
4	Citric acid	1131.33 ± 9.58ab	1213.74 ± 59.98a	965.81 ± 166.44b
5	Quininic acid	37.49 ± 4.79b	22.24 ± 1.77c	50.68 ± 3.64a
6	Palmitic acid	38.62 ± 2.57a	20.23 ± 0.16b	nd
	Sum	1446.81 ± 40.28a	1444.61 ± 48.07a	1399.60 ± 227.91a
Sugars				
7	Xylose	57.18 ± 0.58a	16.76 ± 1.28b	4.47 ± 0.77c
8	Arabinose	5.39 ± 0.10b	15.11 ± 0.81a	2.36 ± 0.39c
9	Fructose	14,259.18 ± 237.19a	12,389.95 ± 1357.50b	11,731.42 ± 622.19b
10	Mannose	155.25 ± 17.50a	139.88 ± 11.24a	162.82 ± 27.27a
11	Sorbose	nd	nd	54.88 ± 1.50a
12	Glucose 2,3,4,5,6-pentakis-O-(trimethylsilyl)-, o-methyloxyme, (1Z)-	17,227.71 ± 687.96a	17,073.88 ± 1494.37a	15,731.50 ± 1048.49a
13	Rhamnose	17.51 ± 1.60a	nd	10.55 ± 0.87b
13	Myo-Inositol	1508.79 ± 45.70a	1503.50 ± 31.59a	240.54 ± 9.76b
14	Allose	2.69 ± 0.26a	2.10 ± 0.28b	nd
15	Sucrose	21,362.29 ± 1880.50a	21,537.81 ± 1705.67a	22,017.88 ± 849.67a
16	Turanose	223.49 ± 34.64a	160.54 ± 14.71b	17.18 ± 2.04c
17	Sum	54,819.49 ± 2906a	52,839.53 ± 3493.60a	49,973.6 ± 2478.50a
Alcohol				
18	Scyllo-Inositol	64.63 ± 3.99c	116.81 ± 1.38b	190.91 ± 9.79a
	Total	56,330.92 ± 2950a	54,400.95 ± 3463.03a	51,564.11 ± 2695.72a

Interestingly, 3 compounds showed some hereditary differences in OCJ (oxalic acid, sorbose and rhamnose). Among them, oxalic acid and rhamnose were undetectable only in OCJ, which caused obvious discrepancies between OCC and its donors. However, sorbose was undetectable in both OCJ and its layer source donor O.

### Volatile compositions of OCC and its donors

With regard to the volatiles in the peels of the three cultivars, 36 substances are listed in Table 4, including monoterpenes, sesquiterpenes, alcohols, aldehydes, phenols and others. The monoterpenes were the most abundant profiles quantified; *d*-limonene was the dominant compound, accounting for 88.65, 81.23 and 80.77% of the total volatiles in OP, OCP and CP, respectively. After *d*-limonene, the main common compounds in the three cultivars were *γ*-terpinene, *β*-myrcene and *α*-pinene.

**Table 4:** Volatiles profiles (μg g^−1^ FW) in the peels of OCC and its donor plants

No.	Volatiles (μg g^−1^)	OP	OCP	CP
Monoterpene				
1	*α*-Thujene	144.35 ± 27.72b	207.23 ± 30.8a	264.98 ± 30.08a
2	α-Pinene	804.57 ± 153.03b	842.83 ± 125.46ab	1089.03 ± 117.82a
3	Sabinene	128.77 ± 24.57a	140.56 ± 22.06a	173.87 ± 18.41a
4	*β*-Pinene	236.92 ± 45.00b	429.98 ± 66.59a	521.91 ± 59.04a
5	*β*-Myrcene	1262.26 ± 244.69a	1204.28 ± 188.37a	1506.58 ± 172.6a
6	*α*-Phellandrene	33.08 ± 7.50c	55.34 ± 6.54b	70.20 ± 5.31a
7	*α*-Terpinene	73.05 ± 13.42b	118.21 ± 17.05a	143.5 ± 15.66a
8	*d*-limonene	60,800.80 ± 7185.92a	59,857.96 ± 5806.31a	66,345.79 ± 5599.97a
9	*β*-cis-Ocimene	46.03 ± 8.01	49.36 ± 8.11b	68.38 ± 7.56a
10	*γ*-Terpinene	3213.21 ± 583.21b	5430.11 ± 835.11a	6360.18 ± 679.34a
11	Terpinolene	149.98 ± 29.22b	246.07 ± 38.77a	286.81 ± 31.14a
Monoterpene alcohols				
12	Linalool	152.41 ± 22.04a	55.37 ± 5.15b	54.97 ± 5.28b
13	*α*-Terpineol	76.01 ± 7.88a	64.6 ± 5.69a	40.38 ± 31.29a
Monoterpene aldehydes				
14	Citronellal	28.25 ± 5.06a	19.82 ± 3.2b	19.28 ± 1.00b
Monoterpene esters				
15	Methyl 2-methyloctanoate	227.59 ± 0.78a	225.77 ± 2.46a	228.57 ± 0.64a
16	Citronellol acetate	5.56 ± 0.46b	16.83 ± 3.03a	15.44 ± 1.75a
17	(*R*)-lavandulyl acetate	16.55 ± 3.36c	68.04 ± 10.32b	98.38 ± 11.36a
	Sum	67,399.39 ± 8348.50a	69,032.36 ± 7166.67a	77,288.24 ± 6729.2a
Sesquiterpene				
18	Copaene	44.00 ± 8.93b	60.57 ± 10.44ab	68.01 ± 7.69a
19	*β*-Cubebene	35.07 ± 7.50b	45.92 ± 7.44ab	52.66 ± 5.54a
20	*β*-Elemene	21.77 ± 3.73a	18.42 ± 3.55a	25.39 ± 3.63a
21	Caryophyllene	22.68 ± 3.81b	58.03 ± 9.62a	68.36 ± 7.67a
22	(*E*)-β-Famesene	37.4 ± 7.50b	117.74 ± 23.03a	128.65 ± 12.12a
23	Germacrene D	123.72 ± 26.64b	2772.96 ± 488.7a	2525.56 ± 267.24a
24	*γ*-Elemene	17.19 ± 2.72b	196.80 ± 35.29a	174.66 ± 18.57a
25	(−)-*β*-Elemene	440.45 ± 93.78a	138.93 ± 24.83b	171.49 ± 19.39b
26	*δ*-Cadinene	57.02 ± 12.36a	69.14 ± 12.49a	79.21 ± 9.92a
27	*δ*-EIemene	33.08 ± 6.31c	155.35 ± 26.62b	195.32 ± 19.83a
Sesquiterpene alcohols				
28	Nootkatone	2.79 ± 0.96c	28.59 ± 3.67b	67.88 ± 4.92a
	Sum	835.19 ± 172.72b	3662.44 ± 645.18a	3557.22 ± 375.92a
Alcohol				
29	(*E*)-3-Hexen-1-ol	8.30 ± 1.13c	34.14 ± 2.09a	15.79 ± 3.19b
Aldehydes				
30	3-Hexenal	51.52 ± 4.72c	72.66 ± 1.12a	64.51 ± 3.14b
31	Hexanal	25.21 ± 2.49a	25.38 ± 0.26a	18.56 ± 1.05b
32	(*E*)-2-Hexenal	5.49 ± 1.13ab	7.48 ± 1.5a	4.41 ± 1.09b
33	Decanal	72.59 ± 13.82a	80.43 ± 15.23a	99.15 ± 10.55a
	Sum	163.11 ± 20.83a	220.10 ± 16.89a	202.42 ± 18.21a
Phenol				
34	2,4-di-t-butylphenol	nd	42.13 ± 6.79a	37.71 ± 8.4a
Others				
35	o-Cymene	103.06 ± 19.73b	91.13 ± 12.45b	156.95 ± 18.41a
36	n-Hexadecanoic acid	58.71 ± 15.95b	115.47 ± 39.42b	207.42 ± 33.32a
	Sum	161.77 ± 20.18b	248.722 ± 58.28b	402.08 ± 48.20a
	Total	68,559.46 ± 8553.26a	73,163.61 ± 7884.82a	81,449.95 ± 7136.32a

The results showed that OCP had a stronger correlation with CP than with OP. First, according to the statistical analysis, 25 volatiles were not significantly different between OCP and CP, but only 16 volatiles were not significantly different between OCP and OP. This finding indicated that CP had the dominant position in the regulation of the chemical profiles in OCP and that more chemical traits in OCP were inherited from CP than from OP. Second, the main volatiles in OCP were completely consistent with those in CP, including *d*-limonene, *γ*-terpinene, germacrene D, *β*-myrcene and *α*-pinene (sorted from high to low concentrations), but the relative concentrations of the main volatiles in OP were divergent (*d*-limonene, *γ*-terpinene, *β*-myrcene, *α*-pinene, *β*-elemene). This was mainly because the content of germacrene D was significantly higher in OCP and CP than in OP, strongly suggesting that germacrene D mainly originated from CP and that OP had less impact on the development of OCP. Third, it is worth noting that 2,4-di-t-butylphenol was truly unique and was only undetected in OP; but possessed by both OCC and C.

In addition, most of the volatiles in OCP were either inclined to one donor or maintained some degree between the two donors. However, only (*E*)-3-hexen-1-ol and 3-hexenal were significantly increased in OCP compared with the two donors.

In the edible juice sacs, up to 19 volatile compounds were detected (Table 5). OCJ was highly correlated with OJ in total volatiles and monoterpenes (the leading volatiles), especially in dominant substances; the concentration of *d*-limonene in OJ and OCJ was significantly higher than that in CJ, representing 78.07 and 72.64% of the total volatiles in OJ and OCJ, respectively, but only 60.03% of that in CJ. In addition to *d*-limonene, significant similarities in methyl nonanoate, copaene, and octanal were also observed between OJ and OCJ, and we hypothesized that all these compounds in OCJ originated from O to a great extent.

**Table 5:** Volatiles profiles (μg g^−1^ FW) in juice sacs of OCC and its donor plants

No.	Volatiles (μg g^−1^)	OJ	OCJ	CJ
Monoterpene				
1	Linalool	46.22 ± 2.08a	33.91 ± 1.31b	20.53 ± 2.07c
2	*γ*-Terpinene	20.90 ± 0.94b	16.01 ± 1.74c	28.08 ± 3.62a
3	*d*-Limonene	383.05 ± 32.38a	402.18 ± 24.76a	298.7 ± 10.03b
4	*β*-Myrcene	44.59 ± 0.86a	36.43 ± 2.91b	29.54 ± 3.83c
5	*β*-Elemene	1.96 ± 0.16c	2.48 ± 0.08b	3.09 ± 0.42a
Monoterpene esters				
6	Methyl 2-methyloctanoate	11.01 ± 0.55b	18.12 ± 0.56a	1.80 ± 0.04c
7	Methyl nonanoate	1.85 ± 0.05b	1.87 ± 0.04b	3.65 ± 0.38a
	Sum	509.57 ± 37.02a	510.99 ± 31.39a	385.39 ± 20.39b
Sesquiterpene				
8	Germacrene D	0.17 ± 0.03b	2.81 ± 0.48a	0.46 ± 0.06b
9	Copaene	14.31 ± 2.03b	19.92 ± 2.70b	49.69 ± 2.14a
10	*α*-ylangene	nd	16.05 ± 3.59a	nd
11	Germacrene B	11.45 ± 1.80a	6.99 ± 1.95b	0.74 ± 0.19c
Sesquiterpene alcohols				
12	Nootkatone	1.13 ± 0.68b	3.62 ± 1.35a	0.45 ± 0.24b
	Sum	27.06 ± 4.55c	49.39 ± 10.08b	51.34 ± 2.64a
Aldehydes				
13	Decanal	1.47 ± 0.08c	2.47 ± 0.75b	3.59 ± 0.39a
14	Dodecanal	2.25 ± 0.07ab	2.83 ± 0.66a	1.72 ± 0.53b
15	Pentadecanal	2.28 ± 0.16b	3.63 ± 0.74a	2.53 ± 0.54b
16	Octanal	1.06 ± 0.06a	1.13 ± 0.15a	0.69 ± 0.21b
	Sum	7.06 ± 0.38b	10.06 ± 2.31a	8.53 ± 1.67ab
Phenol				
17	2,4-di-t-Butylphenol	3.53 ± 0.26b	6.24 ± 0.11a	5.42 ± 1.54a
18	n-Tridecan-1-ol	23.51 ± 2.13a	16.12 ± 1.02b	12.68 ± 2.53b
	Sum	27.04 ± 2.39a	22.36 ± 1.14a	18.10 ± 4.07a
Others				
19	n-Hexadecanoic acid	1.39 ± 0.14ab	0.83 ± 0.46b	1.52 ± 0.20a
	Total	572.12 ± 44.48a	593.64 ± 45.38a	464.88 ± 28.96b

Moreover, typical volatile metabolites were observed in OCJ. For example, nootkatone and pentadecanal were present at the largest amounts in OCJ. In contrast, *γ*-terpinene was significantly lower in OCJ than in either of the donors. Furthermore, we were particularly interested in *α*-ylangene, which was only detected in OCJ but not in the two donors, and this volatile has rarely been reported in any citrus species.

### Carotenoid constituents of OCC and its donors

As shown in Table 6, a total of 9 carotenoids were detected in OCC and the two donors. Generally, the contents and types of carotenoids in OCC were very similar to that in C in the peels, while they were intermediate between the donors in the juice sacs.

**Table 6:** Carotenoid content (μg g^−1^ DW) in peel and juice sac of OCC and its donor plants

No.	Carotenoid content (μg g-1)	OP	OCP	CP	OJ	OCJ	CJ
1	Violaxanthin	941.53 ± 42.97a	772.11 ± 54.36b	911.83 ± 37.03a	5.02 ± 1.21a	5.20 ± 1.47a	4.17 ± 0.98a
2	Luteoxanthin	67.33 ± 8.93a	33.24 ± 3.57b	29.94 ± 5.62b	18.04 ± 0.69b	21.79 ± 0.76a	0.58 ± 0.11c
3	Lutein	110.04 ± 8.67a	44.54 ± 5.56b	24.98 ± 1.27c	5.67 ± 0.69a	4.97 ± 0.52a	4.72 ± 0.67a
4	Zeaxanthin	61.40 ± 5.73a	3.36 ± 0.84b	4.63 ± 1.21b	34.39 ± 0.83a	19.97 ± 2.32b	6.62 ± 0.58c
5	*β*-cryptoxanthin	356.81 ± 8.04a	9.07 ± 1.66b	14.65 ± 0.96b	290.73 ± 2.83a	132.74 ± 6.43b	4.39 ± 0.60c
6	*α*-carotene	12.13 ± 1.52a	nd	nd	5.27 ± 0.07a	1.46 ± 0.03b	nd
7	*β*-carotene	15.37 ± 3.01a	5.19 ± 1.23b	1.26 ± 0.18c	14.31 ± 1.28a	4.25 ± 1.09b	0.22 ± 0.11c
8	Phytoene	383.58 ± 9.23a	44.71 ± 3.41b	71.34 ± 6.41b	123.30 ± 4.26a	42.01 ± 2.21b	nd
9	Phytofluene	243.24 ± 13.28a	26.80 ± 3.15c	49.58 ± 3.76b	106.59 ± 0.57a	18.43 ± 2.78b	2.50 ± 0.37c
	Total	2191.43 ± 101.38a	939.02 ± 73.78b	1108.21 ± 56.44b	603.32 ± 12.44a	251.10 ± 17.61b	23.21 ± 3.41c

Notably, donor O had the highest contents of all carotenoid components in both the peels and juice sacs among the three genotypes. The carotenoids, except violaxanthin, lutein and phytofluene, in OCP were all significantly consistent with CP. The carotenoids in OCJ were an intermediate between those in the two donors except of violaxanthin, luteoxanthin and lutein. In fact, all of the carotenoids detected in OJ and OCJ were particularly higher than those detected in CJ. It is remarkable that α-carotene accumulated much less than other carotenoids both in the peels and juice sacs.

The dominant components in the peels and juice sacs of OCC and the two donors were different. Violaxanthin was the primary component in the peels, and *β*-cryptoxanthin was dominant in the juice sacs. The main carotenoids in OCJ, such as *β*-cryptoxanthin, phytoene and phytofluene, changed much more than those in OCP, which maintained the flesh color of OCC similar with layer donor O.

The correlation of the total carotenoids in OCC and in each of the two donors was analyzed to determine the source of tissue coloration. It was suggested that the carotenoid accumulation in OCC had an obvious donor bias and was different in the peels and juice sacs (Table 7). In the peels, the total carotenoids in OCC were significantly correlated with those in C and O. In the juice sacs, only the correlation coefficient between OCC and donor O was statistically significant (0.957). This donor bias in the carotenoids in the peel and juice sac of mature OCC can partly explain why the peel of OCC is light yellow, similar to donor C, whereas the juice sac is dark orange, similar to donor O.

**Table 7:** Correlation coefficient of carotenoid content between OCC and two donors

Tissues	Metabolites	Cultivars	O	OCC	C
Peel	Carotenoids	O	1		
		OCC	0.892^**^	1	
		C	0.905^**^	0.999^**^	1
Juice sac	Carotenoids	O	1		
		OCC	0.957^**^	1	
		C	0.146	0.210	1

### PCA analysis of metabolites in the peels and juice sacs of OCC and the two donors

In terms of the three categories of metabolites, principal component analysis (PCA) was performed to compare the different tissues in OCC and the two donors.

**Fig. 2:**
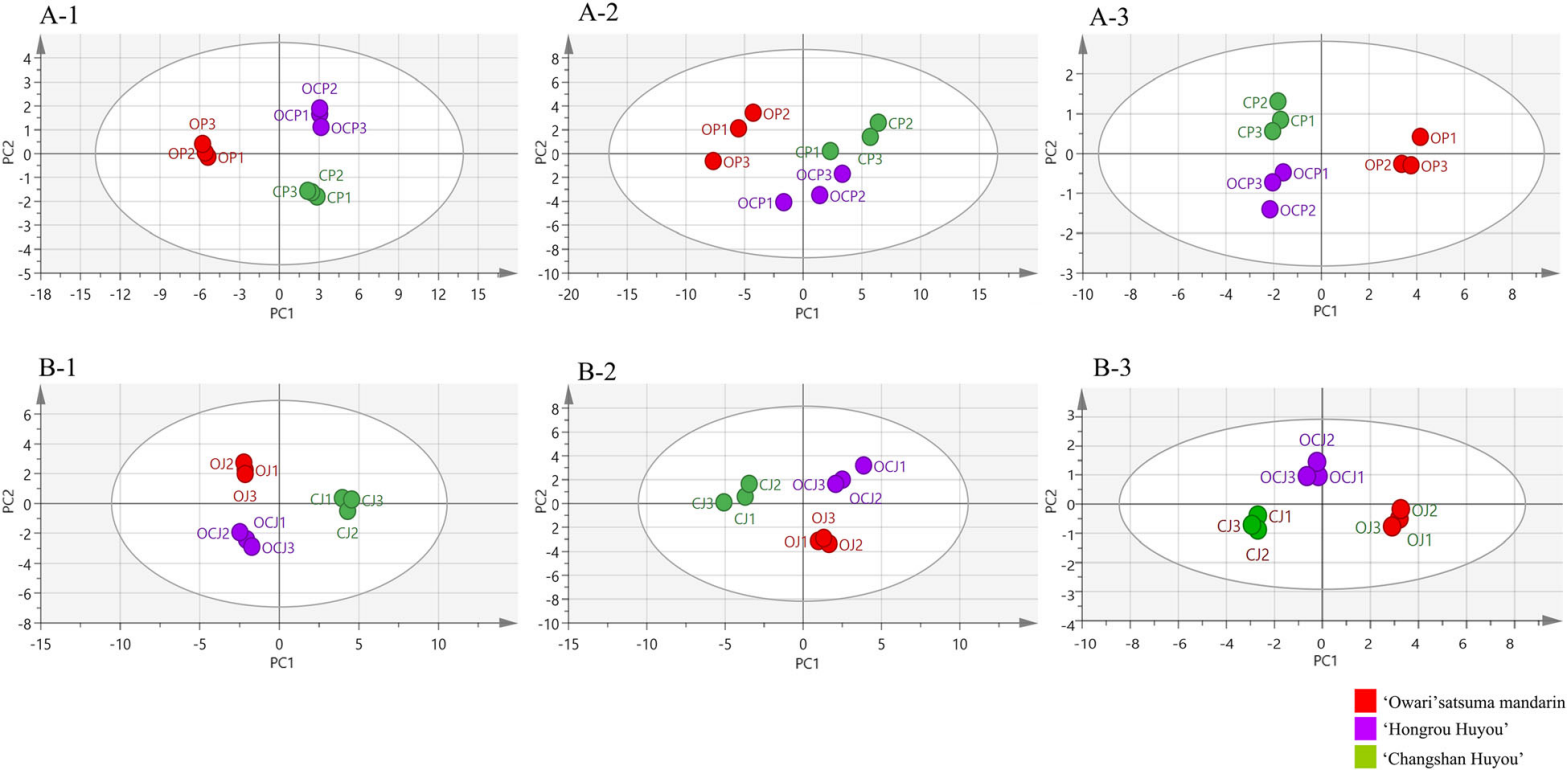
Metabolite contents of the fruit tissues in OCC and its donor plants were analyzed by PCA Primary metabolites (A-1), volatiles (A-2) and carotenoids (A-3) were analyzed in the peels of the three cultivars. Primary metabolites (B-1), volatiles (B-2) and carotenoids (B-3) were analyzed in the juice sacs of the three cultivars. The bold dots colored in red, purple, and green represent O, OCC and C, respectively.

In the PC1 direction of the score map, there was a clear distinction between donor O and the other genotypes (OCC and donor C) in primary metabolites (Fig. 2A-1), volatiles (Fig. 2A-2) and carotenoids (Fig. 2A-3), according to the peels.

In the juice sacs, donor C was clearly distinguished from OCC and donor O in the primary metabolites (Fig. 2B-1), volatiles (Fig. 2B-2) and carotenoids (Fig. 2B-3), according to the PC1 direction of the score map. However, OCJ was separated from OJ in PC1 (Fig. 2B-3), indicating a novel profile of the carotenoid accumulation pattern in the chimera.

## Discussion

Studies focused on phenotypes, fruit qualities and genome compositions [2–5] have contributed to the knowledge of chimeric plants; however, the mechanism of metabolite accumulation in genetically different cells remains unknown. In this work, the profiles of primary metabolites and secondary metabolites were systematically compared in a novel citrus chimera, OCC, and its donor plants, which may provide valuable insight into the genetic contributions and inheritance patterns from grafting donors to chimeras.

### Donor dominant metabolite analysis in OCC

In citrus chimeras, the juice sacs develop from the L1 cell layer, and the peels are derived from the L2 cell layers [1, 2]. In this study, the metabolites in the OCC chimera were more similar to those in C in the peels, but more similar to those in O in the juice sacs, which seemed to confirm the speculated layer origins. Carotenoids are primary nutrients in citrus, and their content and composition vary greatly among citrus varieties [24]. Several reports have focused on the differentiation of citrus genotypes through differences in carotenoid profiles. For example, thirty-two citrus fruits were clearly separated by differences in the*β*-cryptoxanthin content of juice [16]. Similarly, twenty-five citrus genotypes were classified on the basis of *cis*-violaxanthin and *β*-cryptoxanthin in juice [25]. Furthermore, violaxanthin and *β*-cryptoxanthin in the flavedo and juice sac were successfully used to differentiate among 39 citrus genotypes [26]. Herein, three groups were classified according to the amounts of specific type of carotenoids in citrus. Satsuma mandarin represents mandarin cultivars and contained abundant *β*-cryptoxanthin in both the flavedo and juice sac. Oranges are rich in violaxanthin in both the flavedo and juice sacs. Pummelo was separated from oranges and mandarins, as it lacks *β*-cryptoxanthin and violaxanthin [15]. In this study, donor C was documented to be the hybrid of pummelo, orange and/or other citrus species [27–29]. Notably, the primary cell lineage of C includes pummelo and orange, which contain low levels of *β*-cryptoxanthin. In this study, the level of *β*-cryptoxanthin in OCP was as low as that in CP, while OCJ accumulated much more *β*-cryptoxanthin than did CJ. Likewise, a previous study on the citrus chimera Ekuliku revealed that its juice sac was developed from the L1 donor Nankan (*C. unshiu*), and the peel was developed from the L2 and L3 donor Hamlin (*C. sinensis*) [3]. Similarly, the leaf morphology variation of *Brassica* chimeras was only controlled by red cabbage and was reproducible and directional in progenies [30].

Metabolites are first biosynthesized in vivo. Three key genes (*CitPds*, *CitZds* and *CitCrt*) upstream of the carotenoid biosynthesis pathway were reported to be expressed at low levels in a somatic hybrid between *C. reticulata* and *C. limon*, which were biased towards parent lemon, resulting in low carotenoid content in the hybrid [31]. Similarly, a somatic hybrid between ‘Bonnaza’ naval orange (*C. sinensis*) and rough lemon (*C. jambhiri*) showed a similar carotenoid content to that of rough lemon, whose expression patterns of the lycopene ε-cyclase gene (LCYE) and the zeaxanthin epoxidase gene (ZEP) were more similar to those of rough lemon [32]. These scientists believed that the expression of carotenoid genes was not a simple additive effect between parents but rather indicated a certain amount of genomic imprinting, that is, the expression of homologous genes in polyploids biased toward one parent [31, 32]. Herein, it was interesting that the carotenogenesis of the chimera OCC was a balanced representation of the two sets of genetically different cells. It was assumed that in the newly produced chimera OCC, the homologous genes derived from a distinct layer may be selectively expressed in the same metabolic pathway because of changes in DNA methylation that were speculated to be induced during grafting [33] and finally produce the coordinate on expression patterns in each fruit tissue to achieve the coexistence of two sets of genetically different cells.

### Characteristic metabolite analysis in OCC

However, the accumulation of a number of metabolites (including primary metabolites and volatiles) in OCC were specific to the layer source donors; some metabolites deviated far from the profile observed in both donors (i.e., significantly higher or lower than both donors). This observation was similar to two citrus hybrids that exhibited 56 of the 113 volatile profiles in hybrids that were significantly higher or lower than in parents [34]. In this study, the content of germacrene D (Table 5) in OCJ was 6 and 17 times higher than that in CJ and OJ, respectively. The quantities of arabinose were over 3 and 9 times higher than those in CJ and OJ, respectively (Table 3), and this profile has been reported to be a good source of dietary fiber and could be available for juice production [35]. Taken together, the results suggested that the expression levels of genes were altered, possibly due to layer displacement.

Interestingly, a volatile named *α*-ylangene was exclusively detected in the juice sac of the chimera OCC (Table 5). *α*-ylangene is a unique compound that has been rarely reported in any citrus volatile profiles and is a main sesquiterpenoid at the postmaturation stage in grapes [36]. Similarly, a previous study reported that the citrus chimeras NFF and FNN had specific new bands, in addition to the specific bands of the two donor plants, as detected by RAPD analysis, suggesting that the chimeras interacted at the DNA level [2]. Therefore, it was speculated that genetic mutations involved in intercellular movement may be responsible for *α*-ylangene synthesis exclusively in OCC during the development of the chimera. Recently, genetic mutations were suggested to impact the translocation and biological activities of transcription factors (TFs) within a plant [37, 38]. In addition, the heritable variations caused by intercellular trafficking and genetic mutations were extensively studied in chimeras. A grape periclinal chimera ‘Malian’ was derived from cell invasion into L2 to give rise to a spontaneous mutation with bronze flesh [39]. Some studies have reported that berry color variants in grape Pinot can be mapped back to a mutation on a single locus named the “berry color locus”, which encodes four tandem *MYB* transcription factors on chromosome 2 [40–42]. Fernandez and his colleagues investigated the weight reduction in the berry of a grape chimera, which was caused by unusual *VvpI* gene expression in L1, in L2 or in both cell layers, leading to phenotypic variation (fleshless) in progeny [43]. In a peach mutant, a mutated cell carried a PRUPE.6G281100 allele into L2, causing a change in phenotype from flat to round in peach [44].

### Speculation of genetic laws in the metabolites of chimeras

To date, there is limited knowledge available regarding the inheritance pattern of chemical compounds in plant chimeras. The donor bias was a compelling issue in the artificial synthesis of chimeras and in plant breeding. Arguments on the relationship between the chimeric phenotype and the traits of grafting donors have been proposed. It seemed that the stock donor Satsuma mandarin likely acted as the inner layer (L1) donor, with a focus on carotenoid synthesis [4, 5, 45]. Therefore, several novel phenotypes with “red-flesh”, including OCC in this study, were discovered after grafting. Coinciding with these reports, the coloration in the peel and juice sac of OCC was similar to that of the layer source donor; however, the compositions of primary metabolites (such as organic acids and sugars) and volatiles (such as *γ*-terpinene) were partly different from the layer donor and displayed possible “recombination” between layers. Recently, small RNAs and DNA methylation have been considered to be involved in stock-scion interactions to describe genetic variations in graft chimeras. For instance, researchers have found that some conserved miRNAs were differentially expressed in graft chimera (*Brassica juncea* + *B. oleracea*) progeny rTTT (sexual self-crossing of the chimera) and donor plant TTT (*B. juncea*), which may contribute to the changes in the expression of their target genes [30]. Furthermore, in graft chimeras of *Brassica juncea* and *B. oleracea*, sequencing analysis revealed that DNA methylation affects flowering time- and gibberellin response-related gene expression and may lead to phenotypic variations in progenies [6]. Therefore, because OCC possessed metabolites more similar to one donor or an intermediate between both donors, delivery factors that modulate the genes involved in metabolite production, transport and accumulation may be impaired.

## Conclusions

The gene expression pattern and accumulation of primary metabolites, volatiles and carotenoids derived from the peels and juice sacs of OCC and the two donors were systematically investigated and compared. The content and composition of metabolites were different among the genotypes and the tissues. Metabolites specifically present or absent in certain tissues (α-carotene and phytoene) were identified in three genotypes. A clear donor dominant pattern of metabolite inheritance was observed in the different tissues of OCC, indicating that the metabolites in the juice sacs of the chimera were similar to those from the L1 donor O and that those in the peels of the chimera were similar to the L2/L3 donor C. These profiles provide potential chemical markers for genotype differentiation and citrus breeding assessment; moreover, they provide information for donor selection during artificial chimera synthesis.

## Methods

### Plant materials and sampling

The OCC was generated by top-grafting of the scion C and the stock O in 2001, however, it was found recently in our bud mutation investigation in an orchard in Changshan County of Zhejiang Province (China). Recently, OCC was identified to be a grafting chimera in our analysis of the morphological and DNA characteristics of the chimera and the two donors (unpublished data). For commercial production, OCC and the donors (O and C) were separately grafted onto *Poncirus trifoliata* in 2005 and maintained stable morphologies for 12 years under regular management. Three individual trees were selected for each genotype, and 10 fruits with uniform size, peel color and location on the tree were harvested from each tree at the full ripening stage. Peels including the epidermis, flavedo and albedo were separated carefully and quickly from the juice sacs of each genotype by girdling. The peels and juice sacs obtained from one tree were separately blended and ground into powder in liquid nitrogen. Finally, the samples were preserved at − 80 °C for subsequent research.

### Primary metabolites and volatile extraction

The primary and volatile substances were evaluated using a modification of the procedure originally developed [46]. To determine the primary contents, we first ground 0.2 g of tissue into powder using liquid nitrogen and then added 2.7 ml of pure, precooled (− 20 °C) methanol. These components were mixed, and 0.3 ml of ribitol (0.2 g/ml) was added as an internal standards. The procedure was later applied to the volatile samples.

For volatile analysis, the samples were freeze-dried with a vacuum freeze-drier (Labconco FreeZoneR, USA) and fully ground in liquid nitrogen. A 0.2 g sample of powder was poured into a centrifuge tube (2 ml volume), which was homogenized with 500 μl of double distilled water (DDW) and 500 μl of MTBE (containing 0.02 μl/ml methyl pelargonate), followed by gentle shaking. The samples were vibrated using an ultrasonic bath (model FS60, Fisher Scientific, Pittsburgh, PA) maintained at 4 °C for 40 min and were centrifuged at 12000×g for 10 min at 4 °C. The supernatants (200 μl) were then transferred into another tube. Finally, 1 μl of sample was injected with a syringe and filtered through a 0.22 μm membrane (SCAA-104, ANPEL, Shanghai, China) for gas chromatography-mass spectrometry (GC-MS).

### Primary metabolite and volatile analysis

The compounds were identified by using TRACE GC Ultra GC coupled with a DSQ II mass spectrometer (Thermo Fisher Scientific, Waltham, MA, USA) with a TRACE TR-5 MS column (30 m × 0.25 mm × 0.25 μm; Thermo Scientific, Bellefonte, PA, USA). With pure helium as a carrier gas, the peels (flavedo and albedo) and juice sacs of the samples were assayed at 1.0 ml/min with a split ratio of 50:1 and 1:1, respectively. The concentrations of the primary and volatile substances were calculated as μg/g FW. Three replications were used for each sample.

The public databases Massbank (http://www.massbank.jp/) and Metlin (https://metlin.scripps.edu/index.pCF) supported the identification of tentative metabolite substances; for some other compounds, we obtained information from the published literature.

### Carotenoid extraction

The total carotenoids in OCC and its donor parents were extracted according to a previously described method [47] with some modification. Juice sac powder (1 g) and peel powder (0.5–1 g) were homogenized in a 50 ml centrifuge tube after lyophilization using a lyophilizer (LABCONCO FreeZone®). Next, 15 ml of pigment extraction solvent (n-hexane/acetone/anhydrous ethanol, 2:1:1, v/v/v, containing 0.1‰ BHT) was added. The samples were subjected to ultrasonic vibration for 30 min and centrifuged for 10 min at 4000×g at 4 °C. The supernatants were transferred to another 50 ml centrifuge tube, and the sediment was extracted using 15 ml pigment solvent until it was colorless. The supernatants were combined in a 50 ml separating funnel and washed 3 times using a saturated 10% NaCl solution until neutral, and the underlayer was discarded. Then, the supernatants were separated into a 10 ml centrifuge tube and concentrated under vacuum conditions. The samples were redissolved with 2 ml of methyl tert-butyl ether (MTBE) and 2 ml of 10% KOH (containing 0.1‰ BHT), and the residue was dried under nitrogen. The samples were kept in the dark for 10 h for saponification. Then, 4 ml of saturated NaCl and 2 ml of MTBE (containing 0.1‰ BHT) were added to better separate the layers and to wash away the water, and 5 ml of NaCl was added 3 times to wash the solution to neutral. Meanwhile, the supernatant was concentrated by vacuum and was diluted with 0.6–1 ml of MTBE (containing 0.1‰ BHT). The samples were centrifuged at 12000 rpm for 30 min at 4 °C for subsequent analysis.

### Carotenoid analysis

A gradient elution method of OCPLC, composed of A (acetonitrile/methanol, 3:1, v/v, containing 0.1‰ BHT, 0.05% TEA) and B (100% MTBE, containing 0.1‰ BHT) as the mobile phase, was used to determine the carotenoid contents. The flow rate was fixed at 1 ml/min. The following gradients were used: 0 min, (95:5); 0–10 min, A-B (95:5); 10–19 min, A-B (86:14); 19–29 min, A-B (75:25); 29–54 min, A-B (50:50); 54–66 min, A-B (26:74); and 67 min, A-B (95:5). The volume of the above gradient solvent was 20 μl, and the test adopted an external standard method for quantitation. All carotenoid extraction, saponification and other assays described above were conducted under low light levels or in the dark.

### Statistical analysis

The concentration of each chemical compound is shown as the mean ± standard deviation of three replicates. Statistical analysis was performed using SPSS 19.0 software (SPSS Inc., Chicago, IL, USA). Significant differences were calculated using one-way analysis of variance (ANOVA) followed by Duncan’s multiple-range test at the 5% level (*p* < 0.05) and are shown in the tables with lowercase letters (a, b, c, etc.) between cultivars. Undetectable substances are marked with “nd” in all metabolite profile tables. Correlation analysis was carried out by Pearson’s test, and significant differences were marked with “**” (*p* < 0.01). Principal component analysis was carried out by SIMCA 14.1.

## Data Availability

All data generated and analyzed in this study is presented in this published article.

## Abbreviations

CJ: the juice sac of ‘Changshan Huyou’
CP: the peel of ‘Changshan Huyou’
OCJ: the juice sac of OCC
OCP: the peel of OCC
OJ: the juice sac of ‘Owari’ satsuma mandarin
OP: the peel of ‘Owari’ satsuma mandarin;
